# Immunity with Reference to Some of Its Relations to Surgery

**Published:** 1912-10

**Authors:** Ludvig Hektoen

**Affiliations:** Director of the Memorial Institute for Infectious Diseases; Chicago


### BUFFALO MEDICAL JOURNAL

VOL. LXVIII OCTOBER, 1912 No. 3

### Immunity

### With Reference to Some of Its Relations to Surgery

Being the Harrington Lectures before the Alumni Association of the University of Buffalo, May 29 and 30, 1912.

By LUDVIG HEKTOEN, M. D.
Director of the Memorial Institute for Infectious Diseases
Chicago

(Continued from last month.)

THE FORMATION OF ANTIBODIES UNDER VARIOUS CONDITIONS.

By measurement of antibodies as they appear in the blood after the introduction of antigens, much has been learned of the manner of antibody formation, and the most important facts bearing on this point are shown in the antibody curve. The simplest curve, that obtained after one injection of antigen in a normal animal, shows that for two to three or four days, there is, as a rule, either no change or there may be a fall in the specific antibody if such is already present. At the end of this period of latency new antibody appears. This increases until an acme is reached, which in most cases seems to be about the 10th or 12th day; before long begins the last phase of the curve, namely a gradual return to normal, which may last only a few days or several weeks and even much longer.

Animals, including man, normally carry in the blood small amounts of a large number of antibodies, which possess specific affinities for antigenic substances and which probably do not differ much from the corresponding bodies that arise on immunization. In favor of this conclusion is the fact that the primary fall in antibody in the blood on immunization is specific, that is, affects only the antibodies for the particular antigen injected. This fall, which occurs also on reinjection in previously immunized animals, is regarded as the result of neutralization of the existing antibodies, although the amount of antigen introduced often seems too small to cause the fall in this way.

The height and duration of the simple curve vary depending on the kind and amount of antigen as well as on the place of introduction, and also, of course, on the individual animal. It is important to note that the yield of antibodies does not increase in the same ratio as the antigen is increased. Small amounts of bacteria and other antigens may cause more antibody to form on intravenous than on subcutaneous injection. In animals previously acted on by an antigen, the antibody mechanism may be especially sensitive to that antigen and respond more promptly and fully than in the fresh animal. In this way is explained the quick rise of opsonin, often seen on therapeutic injections of vaccines in chronic infectious conditions, as well as other phenomena of allergy which I shall discuss a little later.

When many injections of antigen are given at intervals more or less variations of the simple curve result. It is the rule in immunization with toxins and bacteria to begin with small quantities and to reinject with increasing quantities, carefully graded to avoid severe reactions, at intervals of a few days for a considerable period. A continued and more or less cumulative production of antibodies may be induced also by daily injections of small and constant doses of antigen. In some cases, however, it seems difficult to secure marked cumulative effects. Animals under the continuous influence of antigens eventually lose the power to produce antibodies, to regain it, if at all, only after periods of rest.

A cellular antigen, like bacteria or other cells, may give rise to lysin, agglutinin, opsonin, and other antibodies at the same time. In some cases these different antibodies appear to describe parallel curves; in other cases there is no such parallelism which would seem to indicate that we are dealing with distinct substances.

When several antigens are introduced at the same time, as occurs in secondary and multiple infection, the amount of antibody produced for a particular antigen probably is less than when that antigen alone is introduced. Secondary infections in typhoid fever, such as pneumonia, depress the agglutinin curve. In dogs pneumonic infection (distemper?) may suspend almost completely the production of antibodies for foreign corpuscles. It would seem from this that the same mechanism is concerned in the production of all antibodies as it probably would be different if wholly distinct cell groups were charged with the production of different antibodies. The observations suggest also that the streptococcus and other infections of eruptive diseases, notably small-pox and scarlet fever, and of tuberculosis, may depend in a measure on the inability of the body to respond freely to the stimulus of more than one antigen at a time; and the aggravation of the primary disease, e. g. tuberculosis, as secondary infection sets in, may be due to depression in the production of antibodies for the primary disease, as illustrated by the suspension of the tuberculin reaction by an attack of measles. Here we may assume with v. Pirquet that the measles antigen suspends the formation of the bodies that cause the tuberculin reaction. Other explanations of these phenomena might be advanced.

In his classical experiments on immunity through inheritance and nursing, Ehrlich showed that certain antitoxins pass over into the milk, and since then antibodies have been found to occur also in transudates and other fluids. Normal antibodies are more concentrated in the blood than in the lymph; traces only occur in the cerebrospinal and pericardial fluids and aqueous humor. The results of Dr. Carlson and myself show that so far as the blood and lymph are concerned the changes in concentration during active immunization of dogs with foreign blood run practically parallel, the concentration in the lymph being always somewhat lower than in the blood. In dogs transfused with blood of dogs actively immunized the antibodies rapidly reach the same relative concentration as in normal and actively immune animals. In inflammatory exudates the antibody concentration may approximate that of the blood

As you know a local increase of antibodies may be secured by artificial hyperemia which is producible by various methods such as mechanical passive congestion as practised by Bier, by poultices, by hot air apparatus, by compresses, etc. Any of these methods may do harm unless carefully regulated so as to avoid stasis thereby leading to nutritional disturbances and preventing the free and continuous access of antibodies to the infected area.

### Where Are Antibodies Formed?

It has been found that after injection of antigens, the spleen, bone-marrow, and lymph nodes show increase of antibodies before the blood which, however, soon gains more and more while the tissues mentioned gain relatively less. The most natural conclusion would seem to be that the antibodies are formed in the blood-making organs, and the results of splenectomy certainly favor this view as it is followed by a lower but typical curve if made shortly before or shortly after the injection of the antigen. Experiments also show that the spleen binds antigen; the injection of splenic pulp from an animal injected with antigen into a fresh animal gives new antibody formation. Loss of blood increases the output of antibodies and this also points to the blood-making organs as their source.

It is claimed that antibodies are produced by tissues at whatever place the antigen is introduced, but there is no convincing experimental evidence that such is the case. No matter how or where antigen is introduced, antibodies appear in the blood whence they rapidly make their way into lymph and other fluids and it is consequently impossible to learn whether antibodies in an exudate are produced by the adjacent tissue. Whether antibodies are produced in the blood itself by leukocytes is also under discussion. Now, if they are formed by and in the blood, it would be reasonable to seek them in animals freely transfused with the blood of animals given an optimum dose of antigen a short time before, but in experiments of this kind on dogs Dr. Carlson and I found that no antibody formed in the transfused animals, the antigen very quickly being fixed by tissues or removed from the blood in some way, and the blood evidently takes no direct part in actual manufacture of antibodies.

Judging from experiments on dogs the function of antibody production is not easily upset by various surgical procedures. Removal of the pancreas, of the stomach, of large parts of the small intestines, and various other operations as well as prolonged anesthesia with ether, do not affect seriously the output of antibodies in animals so treated and shortly afterward injected with foreign blood intravenously. It seems as if the mechanisms for the production of antibodies are firmly established and well protected against certain disturbances. In this connection reference may be made to the interesting observations of Graham to the effect that ether, when given for anesthetic purposes, probably by virtue of its fat-dissolving power, greatly reduces the normal phagocytic power of the blood with respect to streptococci, pneumococci, staphylococci, colon and typhoid bacilli. The ether acts both on the leukocytes and the opsonin, and while it is not possible to prove that ether anesthesia predisposes to infection on account of this action it certainly lies near at hand to believe that such might be the case. Graham also finds that lecithin and olive oil suspend the antiphagocytic effect of ether in the test-tube and that administration of olive oil exercises an inhibiting action in vivo at the same time as it prevents in large measure post-anesthetic nausea and vomiting. On account of their fat-solvent powers, chloroform and alcohol may be assumed to have the same or similar effects as ether.

### The Antibodies in Disease.

In acute bacterial diseases the course of the antibodies that develop in the acute attack resembles altogether that of the antibody curve after a single injection of antigen in a normal animal. This holds good for pneumonia, for erysipelas, for diphtheria, for cholera, for the streptococcus opsonin in scarlet fever, etc. These curves show certain definite relations to the clinical phenomena: During the early stages there is a more or less distinct fall in the antibody; as the symptoms begin to subside the curve rises and usually reaches the highest point a day or two after the acme of the symptoms. This curve is readily affected by complications at the onset of which it usually falls, and in rapidly fatal cases the curve may not return but sink lower and lower (pneumonia, cholera.)

A somewhat different type of curve is given by typhoid fever, at least so far as the agglutinin is concerned, which in most cases appears in the first or the early part of the second week, and usually reaches the height in the third week. In the long latency and late acme of the curve one is reminded more of the curve in animals receiving daily injections than of the curve after a single injection of antigen.

That the content of the blood in antibodies may be increased even in chronic infections is indicated very clearly by the power of the serum in syphillis, in gonococcic infections, and in other diseases also, to cause fixation of complement. The allergic reactions, i. e., the special reaction of the infected body to reintroduction of the substances causing the infection, and familiar to all in the form of the tuberculin reaction, are obtainable in an increasing number of chronic infectious processes; according to the current view as to their nature these reactions also depend on the action of new antibodies. Whether the failure for instance of the tuberculin reaction in late stages and generalized forms of tuberculosis is due to a lack of these bodies is not known. The estimations of the opsonic index show that in chronic infections, notably tuberculosis, the opsonin often seems to fall below normal, being however subject to fluctuations of such wide range as to be of diagnostic significance.

### Vaccine Therapy.

Inoculation or vaccine therapy rests on the belief that it is possible to increase the formation of antibodies in an infected organism by injecting the bacteria or virus, properly prepared, that cause the infection. Naturally the question arises why the bacteria already present are not stimulus enough. In the case of chronic, localized infections, in which vaccine therapy is used with most success, one answer is that the infecting germs are so enclosed and walled off that antigenic substances in sufficient quantities do not reach the centers of antibody formation. That this often is the case is shown by the fact that anything which promotes absorption from the focus of infection, such as artificial hyperemia, massage, and exercise, often causes an increase in the amount of specific opsonin. It has been claimed also that antibodies are produced locally and that consequently the injection of antigen on this account serves to throw new and hitherto unused centers of production into activity. I have pointed out that experimentally it has not yet been possible to obtain any satisfactory evidence that there is any local production of antibodies outside of the bloodmaking organs and the lymph nodes. It seems to me that if it be proved that vaccine treatment as claimed by some really is effective in acute infections such as pneumonia, typhoid fever, puerperal septicemia, in which we may assume that there is abundant supply of antigen, then that effectiveness will have to be explained according to principles of which at present we have no clear conception.

In judging of the actual results of vaccine therapy in acute processes, physicians should bear in mind that it is difficult to prove the efficacy of any therapeutic method applied to diseases that are spontaneously curable and that it is impossible to estimate the real benefit of any treatment in such diseases without careful comparisons with a sufficient number of suitable control cases. That vaccine treatment of various chronic and recurrent localized infections may be of value cannot be doubted. Surely tuberculin carefully given promotes the cure of certain forms of chronic tuberculosis, and the literature is full of reports of excellent results from the use of specific vaccines in various forms of staphylomycosis (furunculosis, acne, sycosis). Good results are also obtainable in infections of the urinary tract, especially those caused by strains of the colon bacillus, although bacteria may persist after the subsidence of the active symptoms. Chronic streptococcus infections are also favorably influenced by vaccine treatment. There is such difference between various strains of colon bacilli and of streptococci that the use of vaccines that may be bought does not promise nearly as well as the use of vaccines prepared from the strains actually causing the infections. Weaver and Tunnicliff have found that in rabbits injection of streptococci killed by suspension in 25 per cent. galactose solution induced more or less protection from living streptococci and also a marked increase in the opsonin for streptococci, these effects being more marked than when streptococci killed by heat are used. In the subacute and chronic streptococcus infections in man the best results seem to be obtained by the injection of homologous cocci killed by indifferent chemical agents. All cases selected for vaccine treatment should receive close study; the precise etiological nature of the infection must be determined, and if possible, pure cultures should be obtained, from which the vaccine may be made. Encapsulated exudates and sinuses maintained by foreign bodies are not suitable for treatment by vaccines without the necessary surgical treatment. The indiscriminate use of "mixed vaccines" which is merely another form of the ready-made, shotgun method is undesirable and unscientific.

I have selected as an illustration of the details of vaccine therapy the treatment of gonococcal infection by the injection of killed gonococci, which appears to have a certain amount of real value. Summarizing briefly the recorded experience, I would say that there is reason to doubt that inoculation has any effect in the typical gonorrhea of the adult, at least not in the acute forms; that in chronic gonococcal vulvovaginitis of young girls the treatment by inoculation in the opinion of those who have followed the results most closely is distinctly preferable to all kinds of local treatment, and that it probably shortens the duration of the discharge; that favorable results have been observed in cases of gonorrheal epididymitis and salpingitis; and finally that in chronic gonococcal arthritis and periostitis, the most stubborn of all gonorrheal complications, the frequency and peculiarities of which only recently have become fully understood, injections of killed gonococci may have a distinctly curative action, a conclusion in which practically all observers agree. As emphasized by Irons, too much must not be expected of this method by way of marvelous cures; its proper use is in conjunction with other general measures like rest, aspiration of joints distended with fluids, massage of the prostate, and other surgical and general hygienic treatment.

The material or vaccine used for inoculation consists of suspensions of gonococci in salt solution, the organisms having been killed by heating to 60° C. for one hour. The gonococci are obtained from cultures on ascites-agar or blood-agar on the surface of which they have been growing for 24-48 hours when they are washed off with small quantities of salt solution. While cultures from the lesions of the patient that is to be injected would seem to be preferable, it often is impossible or impracticable to obtain such cultures, and consequently strains from other sources may have to be used. Very often several different strains of gonococci are mixed into one "polyvalent vaccine." The suspensions are made so that fixed quantities contain definite numbers of gonococci, and the number injected at one time has varied from one to twenty millions in the case of children and from one to three thousand millions in the case of adults; speaking generally the initial dose in children should not exceed five and in adults twenty to fifty millions or so. The injections, which are given subcutaneously or intramuscularly are repeated at intervals of a few days, five to eight, and the number of gonococci injected may be increased gradually as warranted by the condition and reactions of the patient. At present general use is not made of the gonococco-opsonic index as a guide to the frequency and size of the injections because it has been found that the clinical manifestations will suffice for that purpose. Experience indicates that in gonococcal arthritis and tubo-ovarian processes the quantity of vaccine injected should be large enough to cause a mild reaction marked by a slight rise of temperature, a little increase in the pain and tenderness of the lesions, and some malaise, lasting for about 24 hours.

### The Specificity of Antigen-Antibody Reactions.

The word "specific" has been used many times in the foregoing, and as it is meant to designate a characteristic of the phenomena of immunity that is of fundamental importance it may be well briefly to make clear the significance of specific and specificity in immunology. It has been found that a given antibody has a very special affinity for the antigen calling it forth, so that its action, whatever that may be, is obtained in the most pronounced degree with that antigen, there being, as a rule, but little or at least less affinity for similar antigens of different species. This, in the briefest and most general sense, is what specific indicates in immunology, and practically all antibody-antigen reactions, both those that occur in the body and those that are observable in the test-tube, follow this law of specific action. It is true that when certain antibodies are richly developed the action may extend to the corresponding antigens from species more or less closely related to that from which is obtained the immunizing antigen, but nearly always in a much less intense degree than in case of the latter. The specificity of antibody-antigen reactions is dependent on the structure of the protein substances that we are dealing with; and it means that there are differences in the structure of similar proteins of different origin, that proteins of different species in most cases are not alike. It is very interesting that certain proteins of animal origin appear to have no specific relations to species. The most striking example of this kind, in fact the only example that is really well substantiated, is furnished by the crystalline lens: the protein of the lens of one species is not distinguishable from those of other species either by means of the precipitin or the anaphylactic reaction as positive reactions are obtainable under the same conditions with lens proteins of widely separated species and even with the lens of the species from which we obtain the immune serum or the sensitized animal. In this case the specificity depends on organ and not species. Whether other proteins, such as those of the sexual cells and of tumors, possess a similar organ-specificness is not determined.

It is the limitation of antibody action by species that gives diagnostic value to the serum reactions in infectious diseases. It is by virtue of this circumstance that agglutination of typhoid bacilli by the serum, in proper dilution, of a patient means typhoid infection; that the reaction to tuberculin means tuberculosis and to gonococci, gonococcal infection; and that the fixation of complement by the antibody-antigen compound has diagnostic significance. It is unnecessary to mention that the action of antitoxic and other therapeutic serums is strictly specific in this sense. If we wish to prevent any infectious disease by means of inoculation or vaccination we are forced by this law of specific action to inject the bacteria or virus, properly prepared, causing the particular infection we wish to prevent. This aspect of the specificness of infectious diseases was expressed clearly and forcibly by Fuller in 1780, when he wrote: "The pestilence (chicken-pox) can never breed small-pox, nor the small-pox the measles, nor they the crystal or chicken-pox, any more than a hen can breed a duck, a wolf a sheep, or a thistle figs, and *therefore one sort cannot be preservative against any other sort*," and, I would add, in reference to inoculation or vaccine treatment, that generally speaking one sort cannot be curative of another sort. If we wish to increase the antibodies against gonococci in the hope thereby favorably to influence a chronic or subacute gonococcal process, we must inject gonococci, and it seems that in view of possible differences in various strains of gonococci, we may do best if we can inject gonococci from cultures of the very strain causing the infection we wish to treat. In all therapeutic inoculation, just as in preventive inoculation, we must inject the bacteria, causing the particular infection we wish to overcome; hence exact, etiologic diagnosis, in many cases possible only by isolation of the infecting germ, is an absolute prerequisite for successful inoculation.

